# Association between fasting glucose/high-density lipoprotein cholesterol ratio and cardiovascular disease risk in Chinese middle-aged and older adults: a longitudinal study

**DOI:** 10.3389/fcvm.2025.1609891

**Published:** 2025-07-14

**Authors:** Jintao Chen, Liying Yan, Jianhai Chen

**Affiliations:** ^1^ Department of Cardiovascular Medicine, The Second Affiliated Hospital, Jiangxi Medical College, Nanchang University, Nanchang, China; ^2^ Department of Gastroenterology, The People's Hospital of Yongcheng City, Shangqiu, China; ^3^ Department of Cardiovascular Medicine, Jiujiang NO.1 People’s Hospital, Jiujiang, China

**Keywords:** high-density lipoprotein cholesterol, fasting blood glucose, cardiovascular disease, CHARLS, middle-aged and older adults

## Abstract

**Background:**

There is currently no information on the association between the fasting blood glucose/high-density lipoprotein cholesterol ratio (FBG/HDL-C) and cardiovascular disease (CVD) incidence.

**Methods:**

Participants in our study, sourced from the China Health and Retirement Longitudinal Study, were grouped into quartiles by FBG/HDL-C ratio. CVD included self-reported heart disease and stroke. The ability of the FBG/HDL-C ratio to predict CVD was assessed using receiver operating characteristic (ROC) curves. Multivariate Cox regression was used to assess the association of FBG/HDL-C ratios with CVD, and potential nonlinear associations were explored using restricted cubic splines.

**Results:**

During the follow-up period from 2012 to 2018, 1,277 out of 6,995 participants (18.26%) developed CVD. There was a nonlinear association between the FBG/HDL-C ratio and CVD incidence in middle-aged and older adults (*P* for nonlinearity <0.05). Compared to the Q1 of the FBG/HDL-C ratio, the adjusted HRs and 95% CIs for CVD in the Q2 to Q4 were 1.17 (0.98–1.40), 1.41 (1.18–1.68), and 1.56 (1.28–1.90), respectively. ROC curve analysis showed that FBG/HDL-C ratio had the highest diagnostic accuracy for CVD than either FBG or HDL-C alone. Furthermore, incorporating the ratio of FBG/HDL-C into the basic model significantly enhanced the prediction of CVD risk.

**Conclusions:**

We found that FBG to HDL-C ratio was significantly associated with an increased incidence of CVD in middle-aged and older adults. The FBG/HDL-C ratio was shown to be more effective in assessing cardiovascular risk than the use of FBG or HDL-C alone.

## Introduction

Cardiovascular diseases (CVD) are the primary cause of death in China and worldwide, placing a significant burden on healthcare systems and the global economy (1, 2). Therefore, identifying and effectively managing the risk factors for CVD is crucial for the achievement of primary prevention. Atherosclerosis is the key pathological mechanism driving CVD, and its formation and progression are dynamic processes influenced by multiple factors (3). Among these, dysregulated glucose metabolism and lipid abnormalities play critical roles in plaque formation and destabilization (4).

Reduced high-density lipoprotein cholesterol (HDL-C) concentrations and elevated fasting blood glucose (FBG) levels, both hallmarks of metabolic syndrome, have been recognized as independent risk factors for CVD (5–8). Previous studies have shown that reduced HDL-C levels are strongly associated with an increased risk of CVD (6, 8). Similarly, elevated FBG levels are significantly correlated with increasing risk of CVD and CVD-related mortality, regardless of the presence of diabetes (5, 7). It is interesting to note that recent studies have revealed a bidirectional relationship between FBG and HDL-C (9). For instance, elevated FBG can reduce HDL-C levels and impair its function through alterations in the proteome and lipid composition of HDL particles, thereby promoting atherosclerosis (9, 10). Conversely, elevated HDL-C levels can reduce FBG levels and ameliorate insulin resistance by improving insulin secretion and enhancing glucose uptake (9, 11). Given this intricate interplay, relying on a single glycemic or lipid marker may not sufficiently reflect an individual's glucose and lipid metabolism status, nor accurately predict CVD risk. In contrast, the FG/HDL-C ratio, as a composite marker, may offer superior predictive value for CVD risk compared to standalone glucose or lipid measures. Indeed, similar composite markers, such as the inflammation-lipid composite marker (e.g., the monocyte-to-HDL-C ratio and the high-sensitivity C-reactive protein/HDL-C ratio), have demonstrated significant utility in predicting CVD (12, 13). Additionally, non-traditional lipid indices, such as the triglyceride-to-HDL-C ratio (TG/HDL-C), have also been found to be strongly associated with increased cardiovascular risk (14). Therefore, the FBG/HDL-C ratio, as a composite indicator reflecting glucose and lipid metabolism, has thus garnered increasing research interest. For example, a study by Guo et al. demonstrated that the FBG/HDL-C ratio was an effective predictor of adverse outcomes in non-diabetic patients with coronary artery disease (CAD) undergoing percutaneous coronary intervention (PCI) (15). Likewise, Deng et al. discovered that a higher FBG/HDL-C ratio was significantly linked to an increased risk of major adverse cardiovascular events (MACE) and cardiovascular mortality in patients with acute coronary syndrome (ACS) (16). However, despite these findings, the relationship between the FBG/HDL-C ratio and CVD incidence remains underexplored, warranting further investigation to validate its utility.

To address this gap, we recruited participants from the China Health and Retirement Longitudinal Study (CHARLS), a nationwide, population-based, prospective cohort study, to investigate t the relationship between FBG/HDL-C ratios and CVD incidence in middle-aged and older adults. Additionally, we evaluated whether incorporating the FBG/HDL-C ratio into the basic model could improve the ability to predict CVD risk.

### Methods

#### Study design and population

The CHARLS is an ongoing research program of the National Development Research Institute (NDI) with participants from 150 counties in 28 provinces in China (17). The CHARLS cohort study was initiated in 2011, with follow-up surveys conducted every two to three years. To date, four follow-up surveys have been completed (in 2013, 2015, 2018, and 2020). Comprehensive details about the study can be accessed on the CHARLS website. CHARLS was approved by the Biomedical Ethics Committee of Peking University, and each participant provided written consent.

For the current analysis, we utilized data from the 2011 to 2018 waves of CHARLS, including a total of 6,995 participants. The inclusion criteria were as follows: (1) Individuals aged 45 years or older with cardiovascular disease status, FBG, and HDL-C data at the 2011 baseline survey (*n* = 11,268). Exclusion criteria were: (1) Participants with non-fasting venous blood samples (*n* = 928); (2) diagnosis of cancer in 2011 (*n* = 105); (3) diagnosis of CVD in 2011 (*n* = 1,474); and (4) loss to follow-up or missing CVD data during the 2011–2018 period (*n* = 1,766). A detailed flowchart of the participant selection process is presented in Supplementary Figure 1.

#### Assessment of incident cardiovascular disease

CVD was determined based on self-reported information. Each participant was asked two key questions: (1) “Have you ever been diagnosed with a stroke by a doctor?” and (2) “Have you ever been diagnosed with heart disease, such as congestive heart failure, angina, coronary heart disease, or any other heart problem diagnosed by a doctor?” If the answer to either of the above two questions was “yes”, it was considered to have a confirmed diagnosis of CVD (18). Participants were followed up from baseline assessment (2011–2012 CHARLS baseline survey) until the onset of heart disease, or stroke, or until the latest survey in 2018, whichever came first (Supplementary Figure 2).

#### Measurement of the FBG/HDL-C ratio at baseline

Venous blood samples from each participant were collected by trained medical staff from the Chinese Center for Disease Control and Prevention (China CDC), following a standardized protocol after a minimum fasting period of 8 h. The samples were then analyzed at the laboratory of Capital Medical University. The formula used to calculate the FBG/HDL-C ratio was as follows: FBG/HDL-C = FBG (mmol/L) divided by HDL-C (mmol/L) (15, 16). Participants were subsequently divided into four groups based on the quartiles of their FBG/HDL-C ratio.

#### Participants characteristics

Participant characteristics were described based on the 2011–2012 survey. Sociodemographic variables included age, sex, educational level, drinking, smoking, residence, and marital status. Additionally, serum uric acid (UA), high-sensitivity C-reactive protein (hs-CRP), and self-reported chronic conditions, such as hyperlipidemia, diabetes, hypertension, cancer, chronic lung disease, liver disease, and chronic kidney disease, were also documented. In our study, hyperlipidemia was defined as having any of the following: HDL-C < 40 mg/dl, triglycerides ≥150 mg/dl, total cholesterol ≥240 mg/dl, low-density lipoprotein cholesterol ≥160 mg/dl, a self-reported history of hyperlipidemia, or current use of lipid-lowering medications (19). Diabetes was defined as a self-reported history of diabetes, an FBG level ≥7.0 mmol/L, or current use of glucose-lowering medications (20). Hypertension was defined as systolic blood pressure (SBP) ≥140 mmHg, diastolic blood pressure (DBP) ≥90 mmHg, a self-reported history of hypertension, or current use of antihypertensive medications (21). Body mass index (BMI) was calculated by dividing weight in kilograms by the square of height in meters, with obesity defined as a BMI ≥28 kg/m^2^ (22).

#### Statistical analyses

Statistical analyses were conducted using RStudio version 4.2.1 and SPSS version 26.0. A two-sided *P* value of <0.05 was considered statistically significant. Missing data were handled using multiple imputations. Categorical variables were expressed as counts and percentages, with differences evaluated using the chi-square test. Continuous variables were presented as means ± standard deviations (SD), and differences were assessed using analysis of variance (ANOVA). The incidence of CVD events was calculated per 1,000 person-years.

Kaplan–Meier curves were produced to describe CVD cumulative incidence and the log-rank test was used to compare differences between groups. Receiver operating characteristic (ROC) curves were employed to assess diagnostic performance, and areas under the curve (AUC) were calculated to quantify the ability of the FBG/HDL-C ratio, FBG, and HDL-C to predict CVD. Differences in AUCs were compared using the DeLong method to determine whether the diagnostic efficacy of the FBG/HDL-C ratio for CVD risk was superior to FBG alone and HDL-C alone (23). We applied multivariable Cox proportional hazards models to examine the association between the FBG/HDL-C ratio and CVD, with results reported as hazard ratios (HRs) and 95% confidence intervals (CIs). The proportional hazards assumption was examined using Schoenfeld residuals and no potential violations were observed. The *p*-value for the trend was calculated by utilizing the median value within each quartile. Three models were developed for the analysis: Model 1 adjusted for age and sex; Model 2 further adjusted for residence, marital status, educational level, smoking, drinking, and obesity; Model 3 included additional adjustments for hyperlipidemia, diabetes, hypertension, systolic and diastolic blood pressure, comorbidities (such as liver disease, chronic lung disease, and chronic kidney disease), hs-CRP, and UA. Restricted cubic spline (RCS) regression models were also employed to explore the potential nonlinear relationship between the FBG/HDL-C ratio and CVD. To assess the incremental value of adding the FBG/HDL-C ratio to the basic model, analyses were performed using the C-statistic, the net reclassification index (NRI), and the integrated discrimination improvement index (IDI).

We also performed subgroup and interaction analyses to examine whether the association between FBG/HDL-C ratio and CVD risk varied by sociodemographic characteristics (age, sex, smoking, drinking, education level, residence, marital status, and obesity). In addition, two sensitivity analyses were performed: (1) repeating the analysis after excluding participants with missing data, and (2) excluding participants diagnosed with diabetes in 2011 to evaluate whether the relationship between the FBG/HDL-C ratio and CVD risk differed in non-diabetic individuals.

### Results

#### Participants characteristics

Table 1 presents the baseline characteristics by quartiles of FBG/HDL-C ratio (Q1: 2.99 ± 0.45; Q2: 4.01 ± 0.25; Q3: 5.04 ± 0.36; Q4: 8.36 ± 4.68). Totally 6,995 individuals were enrolled in our study, their mean age was 58.16 ± 8.82 years, of which 3,279 (46.90%) were male. There were no significant differences between groups in age, smoking habits, chronic kidney disease, chronic lung disease, and liver disease. Individuals with higher FBG/HDL-C ratios were more likely to live in urban areas and have higher levels of education. Blood pressure, hs-CRP levels, uric acid levels, BMI, the proportion of males, and the prevalence of hypertension, hyperglycemia, and hyperlipidemia increased with increasing FBG/HDL-C ratio.

**Table 1:** Baseline characteristics of participants by quartiles of FBG/HDL-C ratio.

Characteristics	Overall	Quartiles of FBG/HDL-C				*P* value
		Quartile 1	Quartile 2	Quartile 3	Quartile 4	
Participants, *N*	6,995	1,746	1,739	1,760	1,750	
FBG, mmol/L	6.05 ± 1.83	5.28 ± 0.71	5.58 ± 0.65	5.86 ± 0.78	7.46 ± 3.00	<0.001
HDL-C, mmol/L	1.32 ± 0.40	1.80 ± 0.34	1.39 ± 0.18	1.17 ± 0.16	0.93 ± 0.21	<0.001
FBG/HDL-C	5.10 ± 3.11	2.99 ± 0.45	4.01 ± 0.25	5.04 ± 0.36	8.36 ± 4.68	
Age, years	58.13 ± 8.79	58.55 ± 9.21	58.02 ± 8.84	57.88 ± 8.50	58.08 ± 8.61	0.124
Male, *n* (%)	3,279 (46.90)	780 (44.70)	784 (45.10)	835 (47.40)	880 (50.30)	0.003
Rural (vs. urban)^a^, *n* (%)	5,890 (84.20)	1,538 (88.10)	1,495 (86.00)	1,484 (84.30)	1,373 (78.50)	<0.001
Educational level^a^, *n* (%)						<0.001
Illiterate	3,198 (45.80)	882 (50.60)	787 (45.30)	788 (44.80)	741 (42.40)	
Primary school	1,552 (22.20)	378 (21.70)	394 (22.70)	388 (22.10)	392 (22.50)	
Middle school	1,491 (21.30)	324 (18.60)	382 (22.00)	379 (21.50)	406 (23.30)	
High school or above	747 (10.70)	160 (9.20)	176 (10.10)	204 (11.60)	207 (11.90)	
Marital status (*n*, %)						0.015
Partnered	6,294 (90.00)	1,553 (88.90)	1,550 (89.10)	1,583 (89.90)	1,608 (91.90)	
Single	701 (10.00)	193 (11.10)	189 (10.90)	177 (10.10)	142 (8.10)	
Smoking^a^, *n* (%)	2,697 (38.60)	655 (37.50)	658 (37.80)	680 (38.70)	704 (40.20)	0.359
Drinking^a^, *n* (%)	2,212 (31.60)	642 (36.80)	518 (29.80)	544 (30.90)	508 (29.10)	<0.001
BMI^a^, kg/m^2^	23.52 ± 3.80	21.91 ± 3.17	23.11 ± 3.70	24.16 ± 3.81	24.94 ± 3.79	<0.001
SBP^a^, mmHg	131.47 ± 21.17	127.92 ± 20.41	130.36 ± 21.43	133.34 ± 21.30	134.38 ± 20.96	<0.001
DBP^a^, mmHg	76.76 ± 12.07	74.62 ± 12.10	76.07 ± 12.49	77.91 ± 11.62	78.50 ± 11.68	<0.001
hs-CRP, mg/L	2.47 ± 6.62	1.77 ± 4.62	2.25 ± 7.25	2.35 ± 5.44	3.50 ± 8.41	<0.001
UA, mmol/L	263.15 ± 72.43	250.99 ± 67.03	255.74 ± 69.12	267.35 ± 72.94	278.43 ± 77.11	<0.001
Obesity^a^, *n* (%)	667 (11.10)	60 (3.90)	109 (7.20)	221 (14.50)	277 (18.90)	<0.001
Hypertension^a^, *n* (%)	2,624 (37.70)	529 (30.40)	593 (34.30)	721 (41.10)	781 (44.90)	<0.001
Dyslipidemia^a^, *n* (%)	3,209 (46.80)	366 (21.30)	453 (26.60)	872 (50.90)	1,518 (88.50)	<0.001
Diabetes^a^, *n* (%)	970 (14.00)	41 (2.40)	70 (4.00)	168 (9.60)	691 (39.60)	<0.001
Chronic Lung diseases^a^, *n* (%)	564 (8.10)	158 (9.10)	142 (8.20)	131 (7.40)	133 (7.60)	0.288
Chronic Kidney disease^a^, *n* (%)	382 (5.50)	113 (6.50)	89 (5.10)	95 (5.40)	85 (4.90)	0.159
Liver disease^a^, *n* (%)	236 (3.40)	49 (2.80)	76 (4.40)	53 (3.00)	58 (3.30)	0.051

CVD, cardiovascular diseases; FBG, fasting blood glucose; HDL-C, high density lipoprotein cholesterol; DBP, diastolic blood pressure; SBP, systolic blood pressure; UA, uric acid; hs-CRP, high-sensitivity C-reactive protein.

^a^ Missing data: 7 for education level, 2 for smoking, 4 for drinking, 1 for residence, 966 for BMI, 966 for obesity, 32 for hypertension, 143 for dyslipidemia, 46 for diabetes, 20 for Chronic Kidney disease, 7 for Chronic Lung diseases, 26 for liver disease, 968 for SBP, and 968 for DBP.

#### Relationship between FBG/HDL-C ratio and cardiovascular disease risk

During a median follow-up period of 6.4 years, there were 1,277 (18.26%) new cases of CVD were documented. The incidence of CVD among participants in quartiles 1–4 was 21.08, 25.65, 34.45, and 45.15 per 1,000 person-years, respectively. The incidence of heart disease per 1,000 person-years for Q1 to Q4 participants was 16.59, 18.87, and 30.96, and the incidence of stroke was 5.33, 7.68, 11.82, and 16.44, respectively.

**Figure 1:**
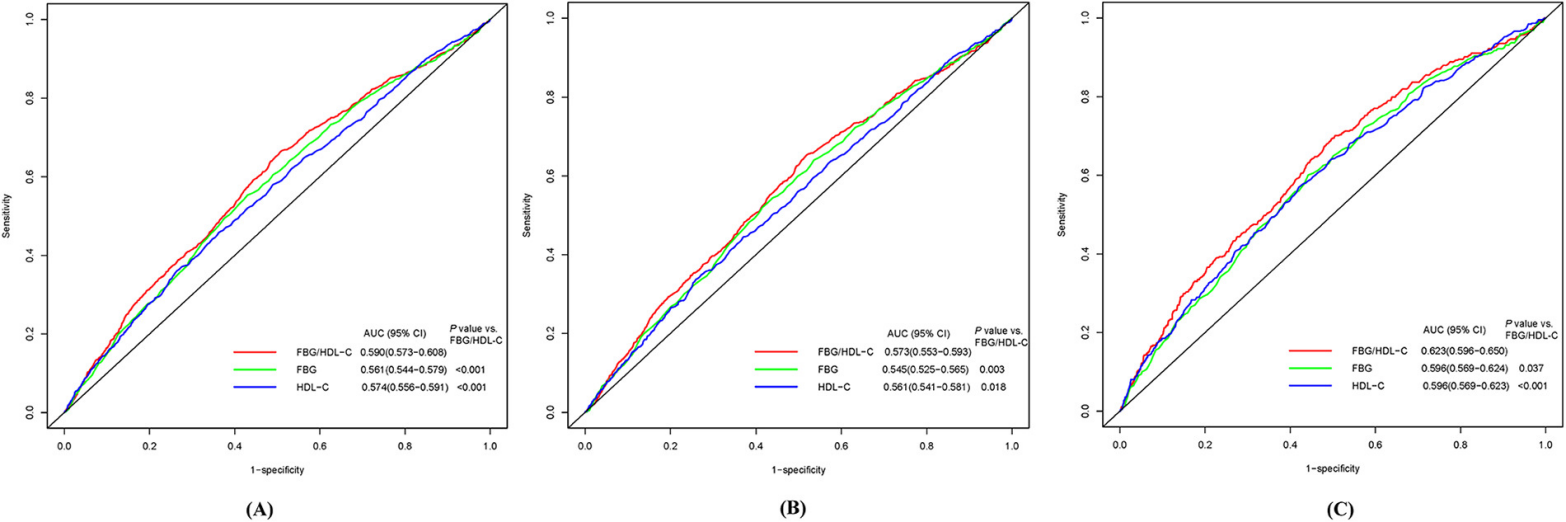
Receiver-operator characteristic curves of FBG, HDL-C, and FBG/HDL-C ratio for predicting CVD **(A)**, heart disease **(B)**, and stroke **(C)**. CVD, cardiovascular diseases; FBG, fasting blood glucose; HDL-C, high density lipoprotein cholesterol; AUC, area under the curve.

The Kaplan–Meier survival curves indicate that the group with higher FBG/HDL-C ratios had a relatively higher cumulative incidence of CVD, stroke, and heart disease (Supplementary Figures 3–5). The ROC curve analysis revealed that the FBG/HDL-C ratio (AUC: 0.590, 95% CI: 0.573–0.608) exhibited the highest diagnostic efficacy for CVD compared to FBG (AUC: 0.561, 95% CI: 0.544–0.579) and HDL-C (AUC: 0.574, 95% CI: 0.556–0.591), with the differences being statistically significant (Figure 1A). Similar to the CVD results, the diagnostic efficacy of FBG/HDL-C ratio was also the highest for heart disease and stroke compared to FBG and HDL-C (Figures 1B,C).

**Figure 2:**
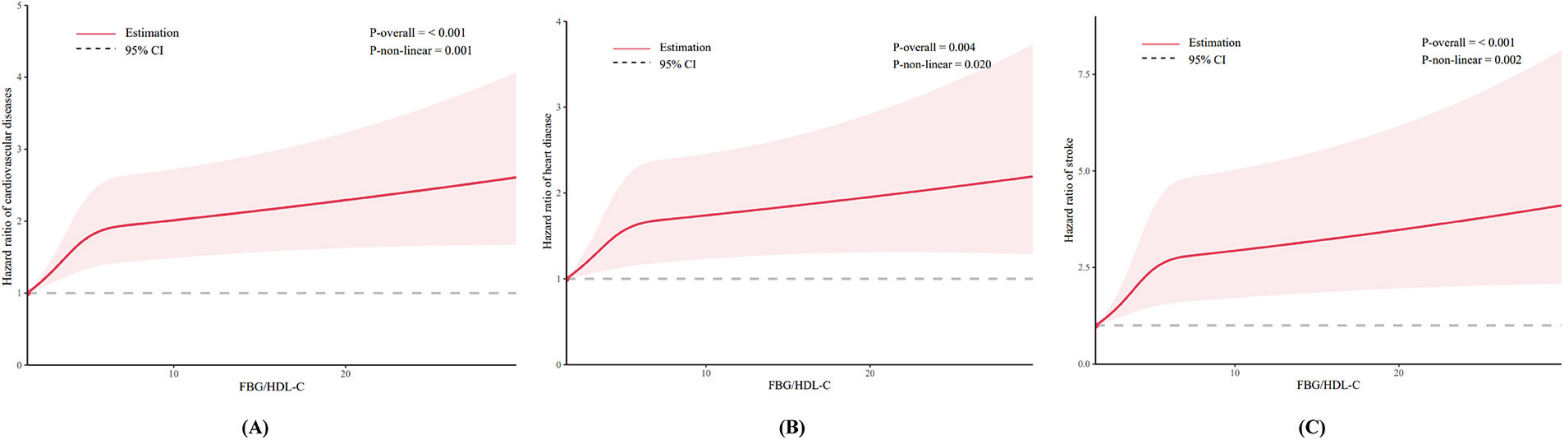
Dose-response relationship between FBG/HDL-C ratio and CVD **(A)**; heart disease **(B)**; and stroke **(C)**.

Table 2 shows the results of Cox regression analyses of the association between FBG/HDL-C ratio and the incidence of CVD. After adjustment for all covariates, higher FBG/HDL-C ratios (continuous variables) were shown to be related to a higher risk of CVD and stroke, with the HRs (95% CIs) of 1.05 (1.00–1.10) and 1.07 (1.00–1.14), respectively. However, no significant association was observed for heart disease (HR: 1.04, 95% CI: 0.98–1.11). Compared to the Q1 of the FBG/HDL-C ratio, the adjusted HRs and 95% CIs for quartiles Q2–Q4 were as follows: for CVD 1.17 (0.98–1.40), 1.41 (1.18–1.68), and 1.56 (1.28–1.90); for heart disease 1.11 (0.90–1.36), 1.31 (1.07–1.60), and 1.47 (1.17–1.85); for stroke 1.35 (0.97–1.89), 1.77 (1.29–2.44) and 1.92 (1.36–2.72) (Table 2). In Figure 2, we utilized an RCS to flexibly model and visualize the predicted relationship between the FBG/HDL-C ratio and incident CVD. The RCS regression model revealed a significant non-linear relationship between the FBG/HDL-C ratio and the risk of developing CVD, heart disease, and stroke (all *P* for overall <0.001 and *P* for non-linearity <0.05) (Figure 2).

**Table 2:** Multivariate Cox regression analysis of the association between FBG/HDL-C ratio and incident CVD.

Categories	Cases, No.	Incidence Rate, per 1,000 Person-Years	Mode 1		Mode 2		Mode 3	
			HR (95% CI)	*P* value	HR (95% CI)	*P* value	HR (95% CI)	*P* value
CVD								
Continues								
Per SD increase	1,277	31.32	1.13 (1.10–1.16)	<0.001	1.12 (1.08–1.15)	<0.001	1.05 (1.00–1.10)	0.036
Quartiles								
Q1	222	21.08	Ref		Ref		Ref	
Q2	265	25.65	1.24 (1.04–1.48)	0.018	1.22 (1.02–1.46)	0.030	1.17 (0.98–1.40)	0.080
Q3	349	34.45	1.68 (1.42–1.99)	<0.001	1.59 (1.34–1.89)	<0.001	1.41 (1.18–1.68)	<0.001
Q4	441	45.15	2.2 (1.87–2.59)	<0.001	2.02 (1.71–2.38)	<0.001	1.56 (1.28–1.90)	<0.001
*P* for trend				<0.001		<0.001		<0.001
Heart disease								
Continues								
Per SD increase	947	22.61	1.11 (1.07–1.15)	<0.001	1.1 (1.06–1.14)	<0.001	1.04 (0.98–1.11)	0.146
Quartiles								
Q1	177	16.59	Ref		Ref		Ref	
Q2	199	18.87	1.16 (0.94–1.42)	0.160	1.13 (0.92–1.38)	0.237	1.11 (0.90–1.36)	0.330
Q3	255	24.38	1.51 (1.24–1.83)	<0.001	1.41 (1.16–1.71)	<0.001	1.31 (1.07–1.60)	0.008
Q4	316	30.96	1.93 (1.60–2.32)	<0.001	1.73 (1.43–2.08)	<0.001	1.47 (1.17–1.85)	0.001
*P* for trend				<0.001		<0.001		0.001
Stroke								
Continues								
Per SD increase	448	10.27	1.13 (1.09–1.17)	<0.001	1.12 (1.08–1.16)	<0.001	1.07 (1.00–1.14)	0.04
Quartiles								
Q1	59	5.33	Ref		Ref		Ref	
Q2	84	7.68	1.48 (1.06–2.06)	0.022	1.49 (1.06–2.07)	0.020	1.35 (0.97–1.89)	0.077
Q3	129	11.82	2.3 (1.69–3.12)	<0.001	2.24 (1.64–3.06)	<0.001	1.77 (1.29–2.44)	<0.001
Q4	176	16.44	3.15 (2.35–4.23)	<0.001	3.08 (2.28–4.16)	<0.001	1.92 (1.36–2.72)	<0.001
*P* for trend				<0.001		<0.001		<0.001

Model 1: adjusted for age, sex.

Model 2: adjusted for age, sex, obesity, education level, marital status, residence, smoking, and drinking.

Model 3: model 2 + further adjusted for hypertension, dyslipidemia, diabetes, chronic lung diseases, liver diseases, chronic kidney disease, DBP, SBP, UA, and hs-CRP.

CVD, cardiovascular diseases; HR, hazard ratio; CI, confidence interval; FBG, fasting blood glucose; HDL-C, high density lipoprotein cholesterol; DBP, diastolic blood pressure; SBP, systolic blood pressure; UA, uric acid; hs-CRP, high-sensitivity C-reactive protein; Ref, reference.

#### Sensitivity analyses and subgroup analyses

Table 3, Supplementary Tables S1, and S2 present the association between the FBG/HDL-C ratio and the risk of CVD, heart disease, and stroke, stratified by sociodemographic characteristics. In most subgroups, the association between the FBG/HDL-C ratio and CVD incidence remained consistent with the primary findings, with no significant interactions observed. In the sensitivity analysis, consistent results were produced when the complete data analysis was performed (Supplementary Table S3). The association between the FBG/HDL-C ratio and CVD in the non-diabetic population did not change after excluding participants with diabetes at baseline (Supplementary Table S4).

**Table 3:** Association of the FBG/HDL-C ratio with CVD risk stratified by sociodemographic characteristics in model 3.

Subgroup	Quartiles of FBG/HDL-C ratio, HR (95% CI)				*P* for interaction
	Quartile 1	Quartile 2	Quartile 3	Quartile 4	
Age					0.566
<60	Reference	1.27 (0.98 −1.64)	1.45 (1.13 −1.87)	1.70 (1.28 −2.25)	
≥60	Reference	1.06 (0.83 −1.37)	1.36 (1.06 −1.74)	1.43 (1.08 −1.89)	
Sex					0.433
Male	Reference	1.06 (0.80 −1.41)	1.37 (1.04 −1.80)	1.73 (1.28 −2.34)	
Female	Reference	1.27 (1.01 −1.61)	1.43 (1.14 −1.80)	1.44 (1.11 −1.87)	
Drinking					0.390
No	Reference	1.10 (0.89 −1.37)	1.32 (1.07 −1.64)	1.50 (1.18 −1.90)	
Yes	Reference	1.34 (0.98 −1.84)	1.63 (1.20 −2.22)	1.68 (1.17 −2.40)	
Smoking					0.860
No	Reference	1.20 (0.96 −1.51)	1.37 (1.10 −1.71)	1.44 (1.13 −1.85)	
Yes	Reference	1.11 (0.82 −1.50)	1.45 (1.08 −1.94)	1.75 (1.26 −2.44)	
Marital status					0.837
Partnered	Reference	1.15 (0.95 −1.40)	1.37 (1.13 −1.65)	1.47 (1.20 −1.81)	
Single	Reference	1.42 (0.83 −2.40)	1.73 (1.02 −2.95)	2.48 (1.35 −4.55)	
Residence					0.653
Rural	Reference	1.15 (0.95 −1.40)	1.35 (1.11 −1.63)	1.57 (1.26 −1.95)	
Urban	Reference	1.40 (0.87 −2.23)	1.90 (1.22 −2.98)	1.68 (1.04 −2.71)	
Obesity					0.759
No	Reference	1.14 (0.95 −1.38)	1.43 (1.19 −1.73)	1.62 (1.31 −2.00)	
Yes	Reference	1.60 (0.83 −3.09)	1.58 (0.86 −2.90)	1.55 (0.83 −2.89)	
Educational level					0.579
Illiterate	Reference	1.19 (0.92 −1.53)	1.39 (1.08 −1.79)	1.42 (1.06 −1.89)	
Primary school	Reference	1.12 (0.77 −1.64)	1.27 (0.88 −1.84)	1.45 (0.96 −2.18)	
Middle school	Reference	1.13 (0.73 −1.75)	1.83 (1.22 −2.76)	1.84 (1.16 −2.92)	
High school or above	Reference	1.50 (0.84 −2.68)	1.34 (0.75 −2.40)	2.30 (1.24 −4.27)	

Model 3: adjusted for age, sex, obesity, education level, marital status, residence, smoking, and drinking, hypertension, dyslipidemia, diabetes, chronic lung diseases, liver diseases, chronic kidney disease, DBP, SBP, UA, and hs-CRP.

CVD, cardiovascular diseases; HR, hazard ratio; CI, confidence interval; FBG, fasting blood glucose; HDL-C, high density lipoprotein cholesterol; DBP, diastolic blood pressure; SBP, systolic blood pressure; UA, uric acid; hs-CRP, high-sensitivity C-reactive protein; Ref, reference.

#### Incremental predictive value of the FBG/HDL-C ratio

The basic model was constructed based on model 3 that included several variables, including age, sex, obesity, marital status, drinking, smoking, residence, education level, hypertension, chronic lung diseases, liver diseases, chronic kidney disease, DBP, SBP, UA, and hs-CRP. Adding the FBG/HDL-C ratio significantly improved the model's predictive power for CVD (C-statistic: 0.641 vs. 0.634, *P* = 0.002), stroke (C-statistic: 0.697 vs. 0.684, *P* < 0.001), and heart disease (C-statistic: 0. 0.645 vs. 0.639, *P* = 0.001) (Table 4). In addition, all continuous NRI and IDI for CVD, stroke, and heart disease were significantly improved (all *P* values <0.05, Table 4).

**Table 4:** Incremental predictive value of the FBG/HDL-C ratio.

Model	C statistic Estimate (95% CI)	*P* value	NRI (continuous) Estimate (95% CI)	*P* value	IDI Estimate (95% CI)	*P* value
CVD						
Basic model	0.634 (0.615–0.653)	Ref	Ref		Ref	
Basic model + FG/HDL-C	0.641 (0.622–0.660)	0.002	0.2172 (0.1578–0.2765)	<0.001	0.0046 (0.0024–0.0069)	<0.001
Heart disease						
Basic model	0.639 (0.620–0.658)	Ref	Ref		Ref	
Basic model + FG/HDL-C	0.645 (0.626–0.664)	0.001	0.1764 (0.1094–0.2433)	<0.001	0.0021 (0.0002–0.0040)	0.028
Stroke						
Basic model	0.684 (0.661–0.708)	Ref	Ref		Ref	
Basic model + FG/HDL-C	0.697 (0.674–0.720)	<0.001	0.3266 (0.2316–0.4215)	<0.001	0.0033 (0.0015–0.0052)	<0.001

The basic model included age, sex, obesity, marital status, drinking, smoking, residence, education level, hypertension, chronic lung diseases, liver diseases, chronic kidney disease, DBP, SBP, UA, and hs-CRP.

CVD, cardiovascular diseases; FBG, fasting blood glucose; HDL-C, high density lipoprotein cholesterol; DBP, diastolic blood pressure; SBP, systolic blood pressure; UA, uric acid; hs-CRP, high-sensitivity C-reactive protein; Ref, reference.

## Discussion

In our study, we observed that the FBG/HDL-C ratio was superior to FBG alone and HDL-C alone in assessing the risk of CVD. Further analyses showed that there was a significant nonlinear association between the FBG/HDL-C ratio and CVD incidence in middle-aged and older adults, with higher ratios predicting a higher risk of CVD. This association remained significant after adjusting for multiple confounding factors. Subgroup analyses were consistent with the primary findings, demonstrating that the association between the FBG/HDL-C ratio and CVD risk was independent of age, sex, smoking, alcohol consumption, residential area, marital status, educational level, and obesity. Sensitivity analyses confirmed the robustness of these results and further indicated that the association persisted even among non-diabetic populations. Additionally, incorporating the FBG/HDL-C ratio significantly improved the predictive power of the baseline model for CVD risk assessment.

To the best of our knowledge, this is the first study to report a nonlinear relationship between the FBG/HDL-C ratio and CVD risk in middle-aged and older adults. Through RCS analysis, we visually demonstrated that a higher FBG/HDL-C ratio is associated with a higher incidence of CVD. An elevated FBG/HDL-C ratio indicates metabolic dysregulation of both glucose and lipids. Specifically, this involves elevated fasting blood glucose and reduced HDL-C, which disrupt the balance between pro-atherosclerotic and anti-atherosclerotic factors, thereby increasing the risk of CVD. Moreover, we found that this combined glycemic-lipid indicator showed greater predictive power for CVD compared to individual markers. This might be due to the complex interplay between glucose and lipids in the body, where a single marker may fail to represent the overall metabolic state and other cardiovascular risk factors. Therefore, composite markers may offer superior predictive value for cardiovascular health prevention and treatment.

These findings enhance our understanding of glycemic-lipid composite markers and highlight their potential advantage in assessing CVD risk in populations without existing disease, an aspect not addressed in previous studies. Prior research has primarily focused on the role of the FBG/HDL-C ratio in evaluating disease severity and prognosis in CVD patients, while our study centers on the relationship between FBG/HDL-C and the risk of CVD in middle-aged and older adults (15, 16). In recent years, there has been growing interest in composite serum biomarkers, including inflammation, glucose, and lipid markers (14, 24–26). For example, the triglyceride-glucose index (TyG), a composite marker of glucose and lipids, has been extensively studied and recognized as an important biomarker of insulin resistance (IR) (27). Recent research indicates that the TyG index not only predicts the occurrence of CVD but can also effectively predict the prognosis of CVD patients (28, 29). Similarly, the atherogenic index of plasma (AIP), a marker derived from the ratio of fasting triglycerides to HDL-C, has recently been identified as a powerful independent predictor of adverse cardiovascular events (30–32). In addition, there are many similar composite serum markers, which are gradually recognized as important tools in predicting CVD. Against this backdrop, our study further explored the FBG/HDL-C ratio as a composite serum marker, and as anticipated, we found a significant relationship between the FBG/HDL-C ratio and the risk of CVD. These findings expand upon previous research by offering new insights into the association between FBG/HDL-C and CVD risk.

We believe that the FBG/HDL ratio plays a crucial role in the mechanism underlying the development and progression of CVD, which can be explained from the perspectives of glucose metabolism disorders and lipid abnormalities. First, glucose metabolism dysfunction is closely associated with the onset of atherosclerosis (4). Hyperglycemia can accelerate the progression of atherosclerosis through multiple pathways, including inducing oxidative stress, exacerbating vascular inflammation, and promoting the formation of advanced glycation end products (AGEs) in the vascular wall and matrix, all of which are key drivers of atherosclerotic lesions (33–35). On the other hand, HDL-C is critical in counteracting the development of atherosclerosis (4). Its mechanisms include promoting reverse cholesterol transport, inhibiting apoptosis, reducing inflammation, and mitigating oxidative stress (36–38). Therefore, elevated levels of FBG together with reduced levels of HDL-C contribute to the accelerated development of atherosclerosis. When FBG and HDL-C are considered in combination, their potential value in the prediction of CVD risk can be more fully reflected.

The main strength of this study lies in our use of a large, nationally representative sample of middle-aged and older adults. However, there are also several limitations. First, due to the observational nature of the study, we cannot establish causal relationships. Nevertheless, FBG and HDL-C have already been widely validated as independent predictors of CVD events. Second, CVD in our study was defined based on self-reported heart disease and stroke, which may introduce classification bias. However, it has been shown in previous studies that self-reports are largely aligned with medical records, which is widely accepted in cohort studies and has been shown to have a negligible effect (39, 40). Third, HbA1c data were not available in this study, which may have influenced the definition of diabetes. Fourth, although we adjusted for multiple cardiovascular risk factors, we cannot completely rule out the possibility of residual or unmeasured confounding bias, which may affect the effect size estimates. Fifth, we did not conduct mediation analysis; if the FBG/HDL-C ratio is a key mediating mechanism through which diabetes or dyslipidemia leads to CVD, adjusting for diabetes and dyslipidemia status in the multivariable Cox proportional hazards model might partially attenuate the effect of the FBG/HDL-C ratio on CVD, thus leading to an underestimation of its true effect (i.e., risk of over-adjustment bias). However, in sensitivity analyses, when we excluded participants with diabetes at baseline, the association between the FBG/HDL-C ratio and incident CVD remained significant. Finally, all participants in our study were from China, which may limit the generalizability of our findings to other countries or regions. Racial differences in glucose and lipid metabolism may exist. For example, existing research suggests that significant differences in insulin sensitivity, β-cell function, and the composition and function of high-density lipoprotein cholesterol (HDL-C) particles may exist between different racial or ethnic groups (41). Therefore, future studies involving different racial populations are needed to confirm these findings.

## Conclusions

In this study, we observed that the ratio of FBG to HDL-C is significantly associated with an increased incidence of CVD events in middle-aged and older adults. The FBG/HDL-C ratio was shown to be more effective in assessing cardiovascular risk than the use of FBG or HDL-C alone. These findings not only provide new evidence for the FBG/HDL-C ratio as an early risk predictor for CVD but also provide new ideas for future clinical CVD screening and risk assessment. In clinical practice, in addition to monitoring independent biomarkers of glucose and lipids, regular monitoring and early intervention should be considered for individuals with higher FBG/HDL-C ratios. This approach may help further reduce CVD incidence in this population.

## Data Availability

The datasets presented in this study can be found in online repositories. The names of the repository/repositories and accession number(s) can be found below: The CHARLS data for our study are publicly available at https://charls.pku.edu.cn/.
