# Supplementary material for: Association between fasting glucose/high-density lipoprotein cholesterol ratio and cardiovascular disease risk in Chinese middle-aged and older adults: a longitudinal study
Source: Front Cardiovasc Med. 2025 Jul 14;12:1609891. doi: 10.3389/fcvm.2025.1609891 (PMC12301364; doi:10.3389/fcvm.2025.1609891)
Supplement: Supplementary file 1 [file Datasheet1.pdf]

Supplementary material

Association between fasting glucose/high-density lipoprotein cholesterol ratio and cardiovascular disease risk in Chinese middle-aged and older adults: a longitudinal study

1. Supplemental Tables

Table S1. Association of the FBG/HDL-C ratio with heart disease risk stratified by sociodemographic characteristics

Table S2. Association of the FBG/HDL-C ratio with stroke risk stratified by sociodemographic characteristics

Table S3. Multivariate-adjusted HR (95% CI) of FBG/HDL-C with CVD among participants after excluding individuals with missing values.

Table S4. Multivariate-adjusted HR (95% CI) of FBG/HDL-C with CVD among participants after excluding individuals diagnosed with diabetes at baseline

.

2. Supplemental Figures

Supplementary Figure 1. The flowchart of the selection of the study population.

Supplementary Figure 2. Study population follow-up timeline.

Supplementary Figure 3. Kaplan–Meier curves for the cumulative incidence of cardiovascular disease.

Supplementary Figure 4. Kaplan–Meier curves for the cumulative incidence of heart disease.

Supplementary Figure 5. Kaplan–Meier curves for the cumulative incidence of stroke.

Table S1. Association of the FBG/HDL-C ratio with heart disease risk stratified by sociodemographic characteristics

Subgroup	Quartiles of FBG/HDL-C ratio, HR (95% CI)				P for interaction
	Quartile 1	Quartile 2	Quartile 3	Quartile 4	
Age					0.566
<60	Reference	1.21 (0.91 -1.61)	1.36 (1.02 -1.80)	1.58 (1.15 -2.17)	
≥60	Reference	0.98 (0.74 -1.32)	1.27 (0.95 -1.69)	1.35 (0.98 -1.87)	
Gender					0.423
Male	Reference	0.93 (0.66 -1.31)	1.39 (1.01 -1.91)	1.77 (1.23 -2.56)	
Female	Reference	1.23 (0.95 -1.59)	1.25 (0.96 -1.62)	1.32 (0.98 -1.76)	
Drinking					0.543
No	Reference	1.01 (0.79 -1.29)	1.27 (1.00 -1.61)	1.35 (1.03 -1.77)	
Yes	Reference	1.35 (0.94 -1.95)	1.39 (0.95 -2.02)	1.78 (1.16 -2.72)	
Smoking					0.878
No	Reference	1.16 (0.90 -1.49)	1.26 (0.98 -1.61)	1.36 (1.03 -1.80)	
Yes	Reference	0.99 (0.69 -1.41)	1.36 (0.97 -1.92)	1.68 (1.13 -2.50)	
Marital status					0.746
Partnered	Reference	1.07 (0.87 -1.33)	1.26 (1.02 -1.56)	1.39 (1.09 -1.76)	
Single	Reference	1.46 (0.77 -2.75)	1.74 (0.91 -3.33)	2.41 (1.15 -5.03)	
Residence					0.928
Rural	Reference	1.09 (0.87 -1.36)	1.29 (1.03 -1.61)	1.52 (1.18 -1.96)	
Urban	Reference	1.25 (0.75 -2.08)	1.58 (0.97 -2.57)	1.39 (0.83 -2.34)	
Obesity					0.593
No	Reference	1.08 (0.87 -1.34)	1.32 (1.07 -1.64)	1.60 (1.25 -2.05)	
Yes	Reference	1.52 (0.74 -3.12)	1.49 (0.77 -2.90)	1.19 (0.60 -2.38)	
Educational level					0.647
Illiterate	Reference	1.08 (0.81 -1.44)	1.24 (0.93 -1.66)	1.33 (0.95 -1.85)	
Primary school	Reference	1.21 (0.77 -1.89)	1.29 (0.83 -2.02)	1.54 (0.94 -2.51)	
Middle school	Reference	0.87 (0.54 -1.40)	1.40 (0.90 -2.19)	1.44 (0.87 -2.39)	
High school or above	Reference	1.93 (0.98 -3.79)	1.86 (0.95 -3.66)	2.55 (1.22 -5.34)	

Model 3: adjusted for age, sex, obesity, education level, marital status, residence, smoking, and drinking, hypertension, dyslipidemia, diabetes, chronic lung diseases, liver diseases, chronic kidney disease, DBP, SBP, UA, and hs-CRP

CVD cardiovascular diseases, HR hazard ratio, CI confidence interval, FBG fasting blood glucose, HDL-C high density lipoprotein cholesterol, DBP diastolic blood pressure, SBP systolic blood pressure, UA uric acid, hs-CRP high-sensitivity C-reactive protein, Ref reference

Table S2. Association of the FBG/HDL-C ratio with stroke risk stratified by sociodemographic characteristics

Subgroup	Quartiles of FBG/HDL-C ratio, HR (95% CI)				P for interaction
	Quartile 1	Quartile 2	Quartile 3	Quartile 4	
Age					0.566
<60	Reference	1.31 (0.79 -2.17)	1.54 (0.95 -2.49)	2.04 (1.22 -3.40)	
≥60	Reference	1.38 (0.88 -2.16)	1.95 (1.27 -2.99)	1.78 (1.11 -2.88)	
Gender					0.722
Male	Reference	1.31 (0.83 -2.09)	1.39 (0.88 -2.18)	1.64 (1.02 -2.65)	
Female	Reference	1.38 (0.85 -2.24)	2.21 (1.40 -3.49)	2.14 (1.28 -3.60)	
Drinking					0.104
No	Reference	1.50 (0.96 -2.35)	1.76 (1.14 -2.73)	2.31 (1.45 -3.67)	
Yes	Reference	1.18 (0.70 -1.98)	1.81 (1.13 -2.92)	1.39 (0.80 -2.41)	
Smoking					0.951
No	Reference	1.43 (0.92 -2.23)	1.82 (1.19 -2.80)	1.95 (1.22 -3.11)	
Yes	Reference	1.22 (0.73 -2.04)	1.66 (1.02 -2.71)	1.76 (1.03 -2.99)	
Marital status					0.990
Partnered	Reference	1.34 (0.93 -1.93)	1.71 (1.21 -2.42)	1.79 (1.23 -2.60)	
Single	Reference	1.64 (0.70 -3.87)	2.22 (0.95 -5.18)	3.03 (1.17 -7.89)	
Residence					0.664
Rural	Reference	1.26 (0.88 -1.80)	1.58 (1.12 -2.23)	1.75 (1.20 -2.54)	
Urban	Reference	2.29 (0.81 -6.47)	3.45 (1.29 -9.25)	3.44 (1.24 -9.56)	
Obesity					0.148
No	Reference	1.34 (0.94 -1.89)	1.83 (1.31 -2.56)	1.74 (1.20 -2.53)	
Yes	Reference	1.59 (0.42 -6.06)	1.56 (0.45 -5.46)	3.36 (0.95 -11.82)	
Educational level					0.137
Illiterate	Reference	1.59 (0.98 -2.60)	2.07 (1.29 -3.33)	1.83 (1.08 -3.10)	
Primary school	Reference	0.88 (0.47 -1.64)	1.29 (0.73 -2.28)	1.42 (0.76 -2.66)	
Middle school	Reference	2.97 (0.99 -8.97)	4.62 (1.59 -13.42)	5.21 (1.70 -15.91)	
High school or above	Reference	0.85 (0.29 -2.46)	0.61 (0.20 -1.80)	1.90 (0.69 -5.20)	

Model 3: adjusted for age, sex, obesity, education level, marital status, residence, smoking, and drinking, hypertension, dyslipidemia, diabetes, chronic lung diseases, liver diseases, chronic kidney disease, DBP, SBP, UA, and hs-CRP

CVD cardiovascular diseases, HR hazard ratio, CI confidence interval, FBG fasting blood glucose, HDL-C high density lipoprotein cholesterol, DBP diastolic blood pressure, SBP systolic blood pressure, UA uric acid, hs-CRP high-sensitivity C-reactive protein, Ref reference

Table S3. Multivariate-adjusted HR (95% CI) of FBG/HDL-C with CVD among participants after excluding individuals with missing values

**Categories**	**Mode 1**		**Mode 2**		**Mode 3**	
	**HR (95% CI)**	**P value**	**HR (95% CI)**	**P value**	**HR (95% CI)**	**P value**
**CVD**						
Continues						
Per SD increase	1.13(1.10-1.16)	<0.001	1.11(1.08-1.15)	<0.001	1.08(1.04-1.13)	<0.001
Quartiles						
Q1	Ref		Ref		Ref	
Q2	1.24(1.04-1.48)	0.018	1.20(0.99-1.45)	0.060	1.18(0.97-1.44)	0.090
Q3	1.68(1.42-1.99)	<0.001	1.56(1.30-1.87)	<0.001	1.42(1.18-1.72)	<0.001
Q4	2.20(1.87-2.59)	<0.001	1.93(1.61-2.30)	<0.001	1.62(1.32-1.98)	<0.001
**Heart disease**						
Continues						
Per SD increase	1.11(1.07-1.15)	<0.001	1.1(1.06-1.15)	<0.001	1.07(1.02-1.13)	0.007
Quartiles						
Q1	Ref		Ref		Ref	
Q2	1.16(0.94-1.42)	0.160	1.11(0.89-1.38)	0.347	1.11(0.89-1.39)	0.368
Q3	1.51(1.24-1.83)	<0.001	1.42(1.16-1.75)	0.001	1.37(1.10-1.70)	0.005
Q4	1.93(1.60-2.32)	<0.001	1.69(1.38-2.08)	<0.001	1.54(1.21-1.94)	<0.001
**Stroke**						
Continues						
Per SD increase	1.13(1.09-1.17)	<0.001	1.12(1.07-1.16)	<0.001	1.09(1.03-1.15)	0.001
Quartiles						
Q1	Ref		Ref		Ref	
Q2	1.48(1.06-2.06)	0.022	1.45(1.03-2.05)	0.035	1.37(0.96-1.95)	0.079
Q3	2.3(1.69-3.12)	<0.001	2.02(1.46-2.80)	<0.001	1.65(1.18-2.32)	0.004
Q4	3.15(2.35-4.23)	<0.001	2.76(2.01-3.79)	<0.001	1.95(1.37-2.79)	<0.001

Model 1: adjusted for age, sex

Model 2: adjusted for age, sex, obesity, education level, marital status, residence, smoking, and drinking

Model 3: model 2+further adjusted for hypertension, dyslipidemia, diabetes, chronic lung diseases, liver diseases, chronic kidney disease, DBP, SBP, UA, and hs-CRP

CVD cardiovascular diseases, HR hazard ratio, CI confidence interval, FBG fasting blood glucose, HDL-C high density lipoprotein cholesterol, DBP diastolic blood pressure, SBP systolic blood pressure, UA uric acid, hs-CRP high-sensitivity C-reactive protein, Ref reference

Table S4. Multivariate-adjusted HR (95% CI) of FBG/HDL-C with CVD among participants after excluding individuals diagnosed with diabetes at baseline.

Categories	Mode 1		Mode 2		Mode 3	
	**HR (95% CI)**	**P value**	**HR (95% CI)**	**P value**	**HR (95% CI)**	**P value**
**CVD**						
Continues						
Per SD increase	1.5(1.35-1.68)	<0.001	1.43(1.28-1.60)	<0.001	1.23(1.07-1.40)	0.003
Quartiles						
Q1	Ref		Ref		Ref	
Q2	1.19(0.98-1.45)	0.079	1.19(0.97-1.45)	0.088	1.14(0.94-1.39)	0.183
Q3	1.80(1.50-2.17)	<0.001	1.73(1.44-2.08)	<0.001	1.58(1.31-1.91)	<0.001
Q4	1.90(1.58-2.28)	<0.001	1.78(1.48-2.15)	<0.001	1.42(1.16-1.75)	0.001
**Heart disease**						
Continues						
Per SD increase	1.4(1.23-1.60)	<0.001	1.31(1.15-1.51)	<0.001	1.16(0.99-1.36)	0.072
Quartiles						
Q1	Ref		Ref		Ref	
Q2	1.10(0.88-1.38)	0.390	1.09(0.88-1.37)	0.428	1.07(0.86-1.34)	0.547
Q3	1.66(1.35-2.04)	<0.001	1.58(1.28-1.94)	0.001	1.49(1.20-1.85)	<0.001
Q4	1.69(1.37-2.08)	<0.001	1.56(1.26-1.93)	<0.001	1.33(1.05-1.69)	0.019
**Stroke**						
Continues						
Per SD increase	1.79(1.51-2.12)	<0.001	1.79(1.50-2.13)	<0.001	1.42(1.14-1.76)	0.002
Quartiles						
Q1	Ref		Ref		Ref	
Q2	1.53(1.06-2.20)	0.023	1.55(1.08-2.23)	0.019	1.41(0.98-2.04)	0.064
Q3	2.23(1.58-3.14)	<0.001	2.23(1.58-3.16)	<0.001	1.83(1.29-2.60)	0.001
Q4	2.68(1.92-3.75)	<0.001	2.7(1.92-3.79)	<0.001	1.78(1.22-2.60)	0.003

Model 1: adjusted for age, sex

Model 2: adjusted for age, sex, obesity, education level, marital status, residence, smoking, and drinking

Model 3: model 2+further adjusted for hypertension, dyslipidemia, chronic lung diseases, liver diseases, chronic kidney disease, DBP, SBP, UA, and hs-CRP

CVD cardiovascular diseases, HR hazard ratio, CI confidence interval, FBG fasting blood glucose, HDL-C high density lipoprotein cholesterol, DBP diastolic blood pressure, SBP systolic blood pressure, UA uric acid, hs-CRP high-sensitivity C-reactive protein, Ref reference

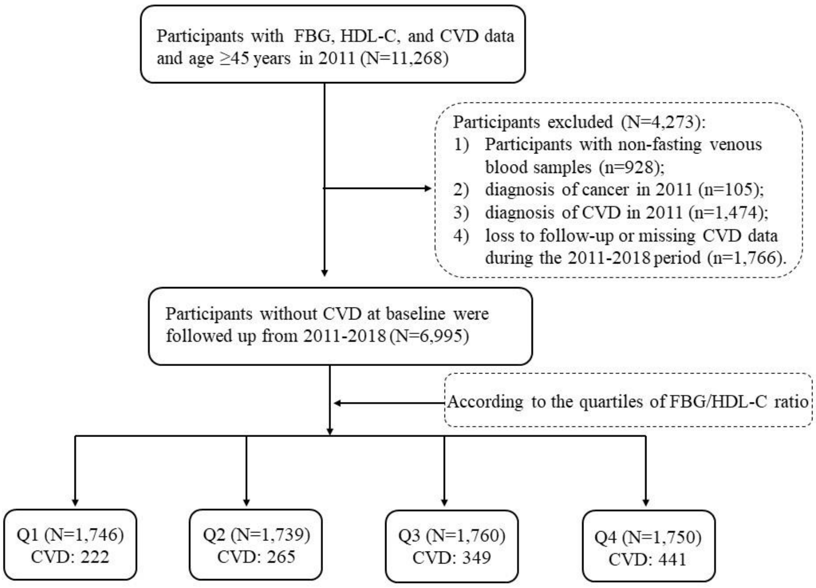

Supplementary Figure 1. The flowchart of the selection of the study population.
Abbreviation: CVD cardiovascular disease, CHARLS China Health and Retirement Longitudinal Study, FBG fasting blood glucose, HDL-C high density lipoprotein cholesterol

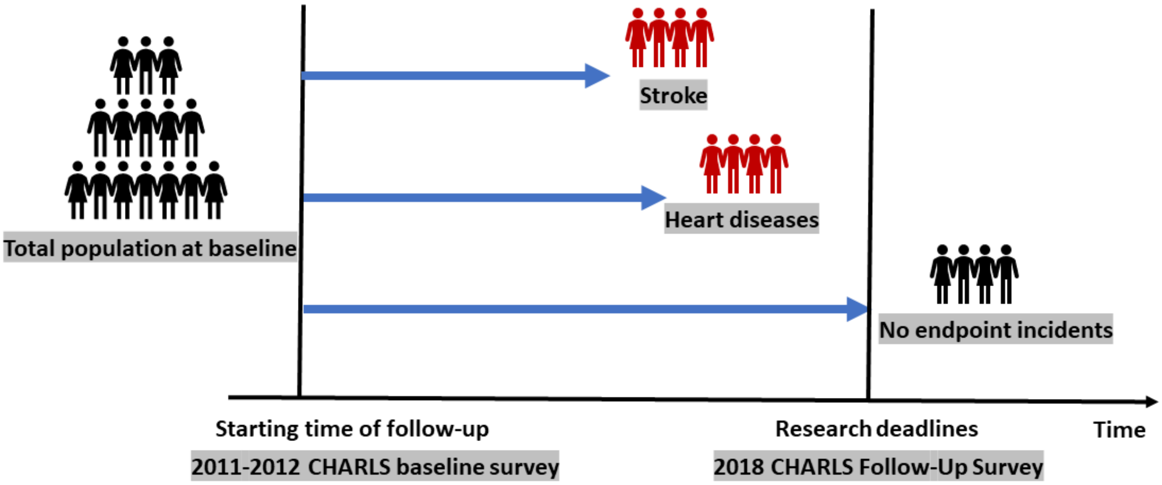

Supplementary Figure 2. The 2011-2012 CHARLS baseline survey calculated the exposure factor (FBG/HDL-C) values and collected coordinating variables, while the 2018 CHARLS follow-up survey assessed endpoint events, with CVD defined as stroke and/or heart diseases.

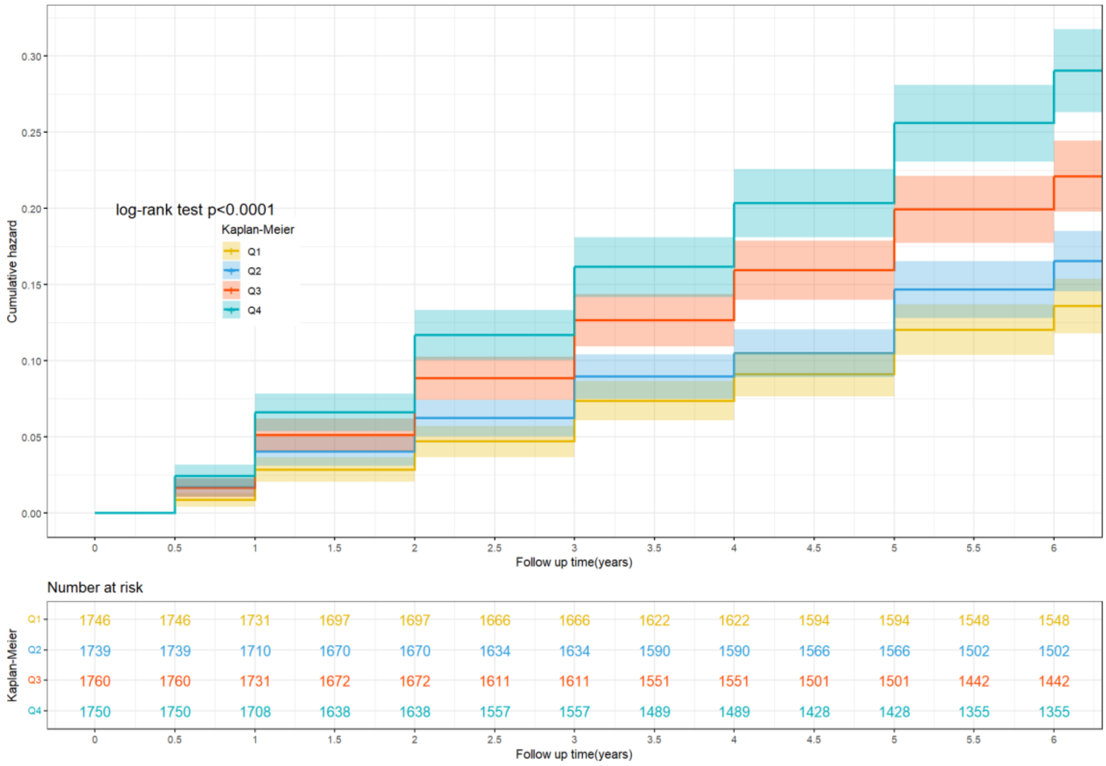

Supplementary Figure 3. Kaplan–Meier curves for the cumulative incidence of cardiovascular disease.

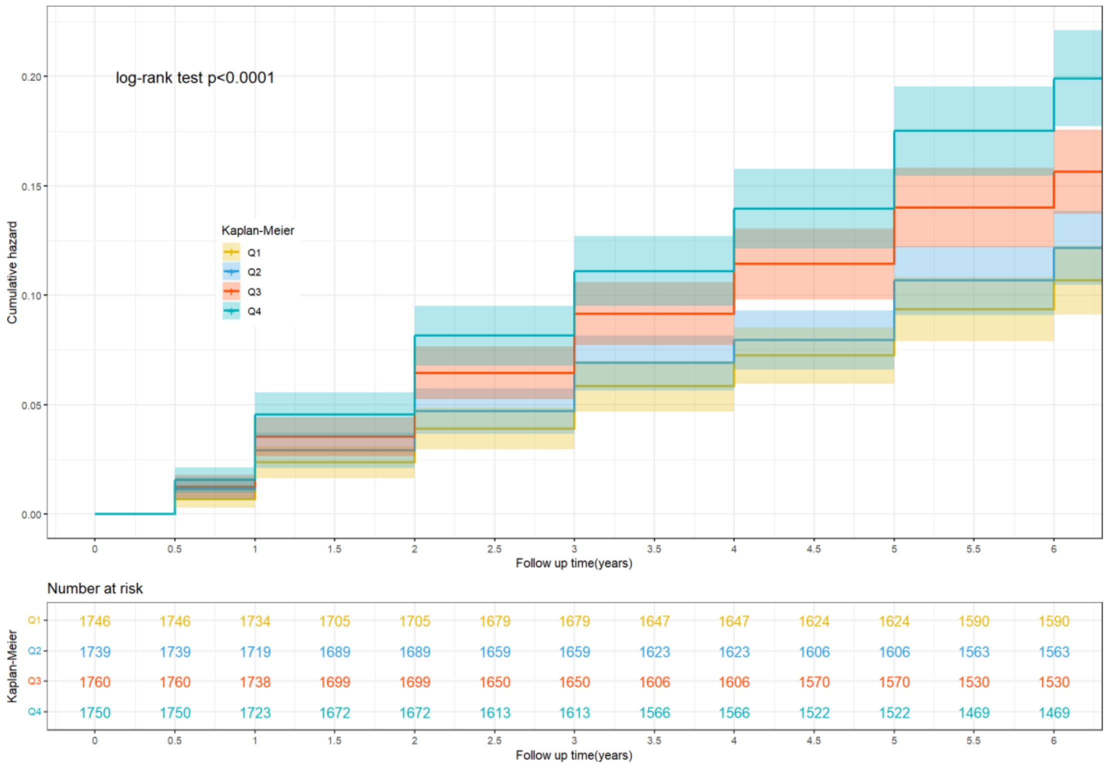

Supplementary Figure 4. Kaplan–Meier curves for the cumulative incidence of heart disease.

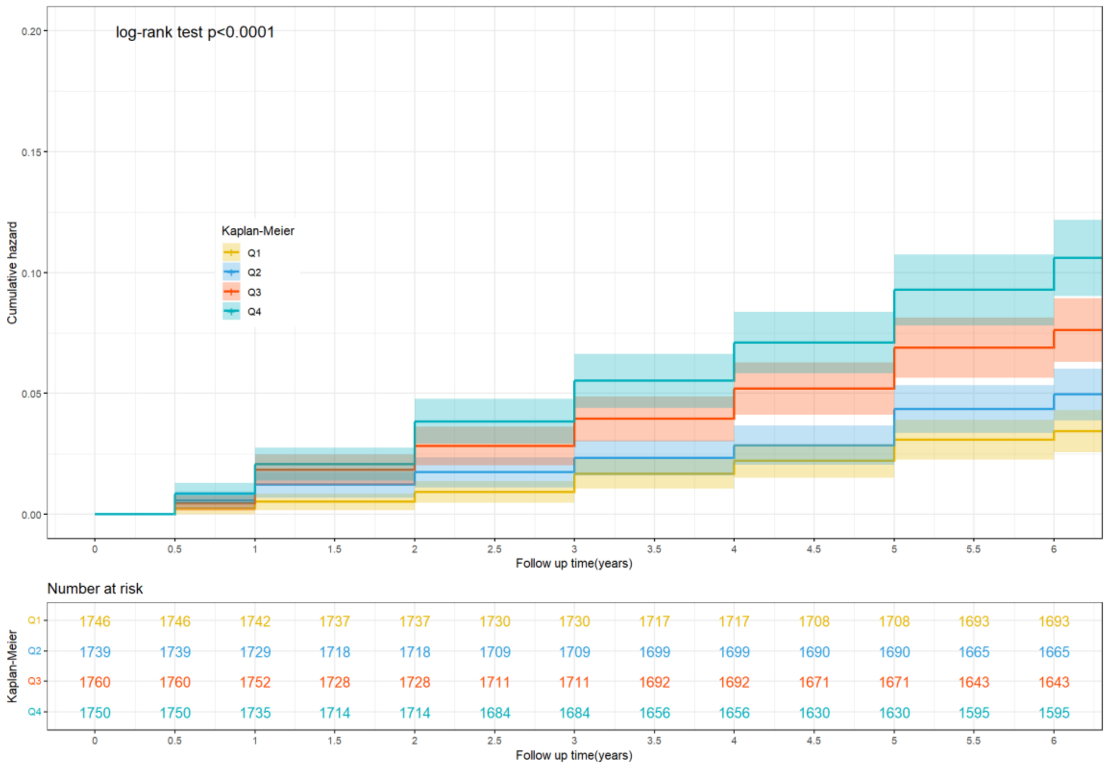

Supplementary Figure 5. Kaplan–Meier curves for the cumulative incidence of stroke.
